# Mapping the cultural divides of England and Wales: Did the geographies of ‘Belonging’ act as a brake on British Urbanisation, 1851–1911?

**DOI:** 10.1371/journal.pone.0286244

**Published:** 2023-05-25

**Authors:** Joseph Day

**Affiliations:** School of Geographical Sciences, University of Bristol, Bristol, United Kingdom; Università Cattolica del Sacro Cuore Sede di Piacenza e Cremona Facoltà di Economia: Universita Cattolica del Sacro Cuore Facolta di Economia e Giurisprudenza, ITALY

## Abstract

Although both the analysis of regional culture and urbanisation are long-standing preoccupations in geography, few studies have considered the relationship between the two, the former traditionally being a topic in cultural geography, while the latter is usually interpreted and analysed as a process in economic geography. Taking evidence from the 1851–1911 censuses of England and Wales, this article analyses individual migration paths to identify stable regions of human interaction by applying a sophisticated community-detection algorithm. By accurately mapping the regions within which the majority of migration occurred between 1851 and 1911 and arguing that the stability of these geographies is evidence of more than just mutable communities but rather of persistent regional cultures, this article responds to previous studies that have sought to identify the cultural provinces of England and Wales. Indeed, by demonstrating that the regions bear a striking resemblance to those that have long been hypothesised as being distinct cultural provinces of England and Wales, this article empirically corroborates their existence. In order to further demonstrate that the regions constitute cultural provinces, this paper incorporates these boundaries into a spatial interaction model (SIM). The results of the SIM not only shows that the boundaries between the regions limited the number of migrants that crossed them–over and above that explained by control variables–and therefore represented the boundaries of cultural provinces, demarcating discrete regions of human interaction–but that such boundaries disproportionately restricted rural-urban migrants, thereby slowing the pace at which England and Wales urbanised. This paper therefore demonstrates that urbanisation should not only be interpreted as only an economic phenomenon, but a cultural one also, and that if urbanisation is to be fully understood, individuals’ attachment to place as a component of their identity, ought to be formally incorporated into models of migration.

## 1. Introduction

The analysis of internal migration in nineteenth-century England and Wales has generally been the domain of economic historians and quantitative geographers—and for good reason. The census has allowed the mapping of both net migration and county-level flows since the pioneering works of both Lawton, and Friedlander and Roshier [1, 2]. While the release of individual-level census data has allowed for more sophisticated methodologies to be employed in the analysis of internal migration, such studies have continued to focus on those determinants which can be easily quantified; specifically, distance and the relative attraction of the settlements which individuals migrated to and from [3]. This has led migration to be conceptualised as a distance-decay function, formalised by spatial interaction models (SIMs) derived from Newtonian gravity models, in which the flows between two objects are reduced to a function of the attraction (estimated from variables such as population and average wage) and distance (conceptualised as Euclidean/Manhattan distance, or the distance in miles or minutes along a road/rail network) between them [4–10]. Although the gravity metaphor remains influential in the conceptualisation and analysis of migration [11, 12], as like interplanetary attraction, larger towns may exert a greater ‘pull’ on migrants compared to smaller towns, unlike interplanetary attraction, terrestrial forces of attraction are mediated by *barriers*. There are no rivers, oceans or mountains in space, whereas on earth, these represent real and meaningful barriers to movement, which can easily be forgotten when conceptualising migration as akin to a gravitational pull [13–15]. If a SIM is to fully capture the frictional costs of migration, it ought to identify and include such barriers as well as distance.

Although the nineteenth century was characterised by rapid urbanisation, this was driven by a minority of migrants travelling relatively short distances upon leaving home or exiting farm/domestic service [16]. That much of the population were relatively immobile might be explained by the concept of ‘belonging’. Across the social sciences, researchers have argued that the need to ‘belong’ is a fundamental societal need, and that the question of self and identity as expressed by the age-old question of ‘who am I?’ cannot be separated from that of ‘where do I belong?’ [17–19] Yet the importance of regional belonging and identity appears to be missing from much of the literature on migration. While some studies have analysed the effect which migration had on individuals’ sense of identity and belonging, none have considered whether individuals’ need to ‘belong’–an inherently geographical concept—might have hindered migration [20–23]. Given the rapidity with which England and Wales urbanised between 1851 and 1911 and the seemingly limitless demand for labour in most British towns and cities, this should be a critical consideration.

However, the determinants of migration and persistent wage inequalities arising from a perpetual imbalance between urban labour demand and supply in nineteenth-century England and Wales have typically been interpreted in neoclassical terms [24–29]; as the product of an imperfect labour market in which the price signal failed to redistribute labour optimally rather than considering non-wage determinants such as social connections or geography [30]. It therefore seems pertinent to consider whether labour markets did not clear in the manner predicted by neoclassical economic theory because of barriers to migration. To use a simple analogy; if the rural and urban ‘pools’ of labour were connected via a stream, the water from the rural to the urban pool was not enough to satisfy demand, resulting in perpetual disequilibrium. Up to now, analyses have assumed this was a consequence of insufficient ‘pressure’–or incentive—to migrate, inhibiting the migration flow from the rural to the urban labour ‘pool’ [27]. This article on the other hand, considers the possibility that a low flow rate, and consequent disequilibrium between the two pools, was not a result of limited pressure, but of *barriers* This article therefore analyses the interdependence between the cultural geographies of belonging and the economic geographies of migration by evaluating the extent to which boundaries separating provincial cultures deterred migration across them.

While it is self-evident that place is an important component of individuals’ identity, defining the location and extent of such ‘cultural provinces’ has thus far proved elusive. Indeed, in order to quantify community structure, what constitutes a community must first be defined. Broadly speaking, communities can either be defined by levels of social interaction, or bonded by common beliefs, interests, etc. Dorling therefore sought to map the historic ‘North-South Divide’ in England, by analysing 75 location-specific attributes across five themes; life expectancy, poverty, education, employment and wealth [31]. Regionalisation algorithms produce spatially discrete units which maximise the similarities between features in the *same* cluster but maximise the differences between features in *different* clusters based on their attributes [32, 33]. However, identifying communities in such a way is profoundly unsatisfying. Commonalities such as language, culture, beliefs etc., are only hallmarks of community because they are evidence of interaction. A group which interacts either forms, or is formed by commonalities. Therefore, interactions imply commonalities, but commonalities do not imply interaction.

Therefore, if communities can only be detected from interactions, long-standing cultural provinces of England and Wales can only be identified from comprehensive evidence of interaction over the long-term. A recent study regionalised human interaction in Great Britain by analysing the connections made over the landline telephone network [34]. While the dataset enjoys comprehensive spatial coverage with over a billion telephone calls, it lacks temporal depth, having been collected over a one-month period, making it difficult to argue that the regions produced represent meaningful, long-standing and stable regions of human interaction, rather than an artefact of the data itself. Historic studies which similarly analyse networks generated by migration and marriage patterns, which despite enjoying good temporal depth, lack comprehensive spatial data. However, unlike modern telephone data, such historic records do not represent a complete network of interactions, and therefore, it is not possible to detect regions using network analysis. Instead, boundaries which were hypothesised to represent meaningful barriers to human interaction were identified. Fox identified the frontier between Leicestershire and Lincolnshire, Bysouth analysed Hertfordshire’s Icknield Way, Carter looked at the boundary between Huntingdonshire and Cambridgeshire, Day examined the boundary that Wiltshire shared with Somerset and Dorset and Phythian-Adams analysed the boundary between Leicestershire and Warwickshire. All five studies found that even when the friction of distance is accounted for, fewer marriages were solemnised between spouses born across county boundaries from one another, than those between spouses born in the same county [35–39]. While these studies appear to have identified meaningful barriers to human interaction, it is by no means evident that these were also the *most* meaningful barriers to human interaction. Numerous studies have used qualitative evidence to hypothesise which boundaries represented the most meaningful barriers to human interaction and why [40–44], but none have been able to prove that watersheds of river drainage basins for example, did indeed form the limits of regional cultures. This study fills this lacuna and resolves the shortcomings of previous attempts to empirically verify the legitimacy of hypothesised boundaries of cultural provinces, by analysing a complete network of human interaction over a sixty-year period. Using the complete, individual-level census returns of England and Wales from 1851 to 1911, this paper reduces the complex network of migration decisions to the primary regions in which most moves occurred. In so doing, it is possible to not only identify a stable geography of provincial cultures, but also show that the boundaries separating them also limited the number of migrants crossing them, especially deterring potential rural to urban migration and thereby slowing the pace at which England and Wales urbanised. This is argued in two sections.

Section 3 details the results of an algorithmic approach to community detection. As migration flows (edges) form a network between places (nodes), the community-detection/network partitioning algorithm is used to group them into ‘communities’, similar to recent analysis of the modern US commuting data [45, 46]. The algorithm groups the ‘nodes’ in the network together to maximise the number of connections (edges) *within* groups and minimise the number of connections/edges *between* groups. The algorithm is run iteratively, grouping the nodes—starting from a perfectly random grouping—until it can no longer make statistically significant improvements to the grouping of the nodes.

However, if the algorithm was *only* trying to maximise the edges between nodes within groups and minimise the edges connecting nodes in different groups, the optimal solution would be to simply put all the nodes into a single group. As this would obviously not be useful, the algorithm is not designed to optimise the proportion of nodes that belong to a single group but rather the ‘modularity’ score. This means the algorithm optimises the communities relative to a perfectly random assignment of nodes to groups [47, 48]. A value of 0 indicates that the regions produced are no better than random, whereas a score of 1 indicates strong community structure.

It is noteworthy that although the community-detection algorithm produces spatially contiguous communities, unlike regionalisation algorithms, no constraint specifying this has been included. That such spatially contiguous regions are produced without specifying them to be so not only confirms Tobler’s first law of geography, but also demonstrates that bounded, internally homogenous regions are meaningful physical and cultural areas which determine individuals’ migration decisions [49]. Therefore, having identified ‘stable’ regions of human interaction between 1851 and 1911, section 4 examines how meaningful these regions were, and the extent to which urbanisation was impeded by migrants’ reluctance to cross such boundaries.

**Fig 1:**
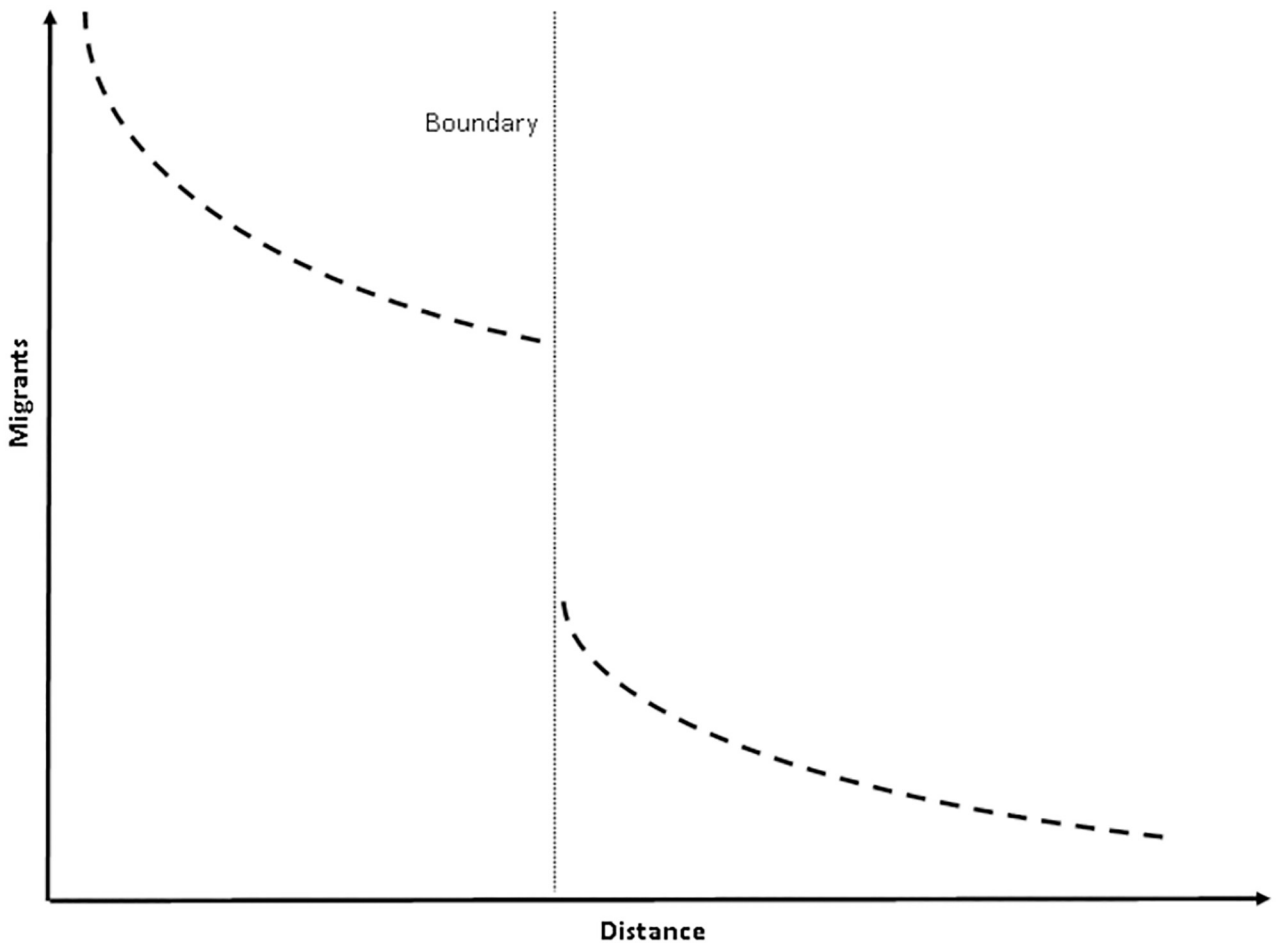
Illustration of the effect a boundary might have on the volume of human interactions. Source: Author’s own illustration.

While it is tempting to assume that the inverse relationship between the volume of migrants and distance remains constant over space—indeed van Lottum goes so far as to suggest that London’s hinterland extends in a perfect circle of radius 136 km from its centre [50]–barriers constitute additional costs to migration, over and above that explained by distance. The effect which such boundaries had on migration can be illustrated by Fig 1. Fig 1 still shows the distance-decay effect, but the addition of a boundary introduces a significant break in the continuity between distance and migrant volumes, resulting in a steep and immediate drop in the number of migrants across a boundary. Consequently, although this study is not the first to consider the effect which barriers may have had on migration, it is the first to identify the most meaningful and enduring breaks in the network of human interaction and quantify the extent to which they impeded urbanisation [51], further demonstrating that the regions identified represent real and meaningful regions demarcating human activity, in other words, cultural provinces.

Using the regions identified in section 3, section 4 analyses their effect on migration in a SIM. This shows that even once other predictors are accounted for, boundaries acted as a significant brake on migration for all groups of migrants, especially rural to urban flows, thus slowing the pace of urbanisation. However, while such regional barriers clearly slowed the transfer of rural to urban labour in the aggregate, such global models do not account for fact that the boundaries between cultural provinces may not have represented an equal barrier to all migrants. Indeed, Fotheringham *et al*. argued that spatially-varying outcomes may not have only been a function of spatially-varying inputs, but of spatially-varying *processes* [52, 53]. In other words, provincial boundaries may have represented more of a barrier to migrants from some places compared to others.

Through these analyses, it can be shown that the boundaries between regional provinces were real and meaningful barriers in peoples’ lives and that the need to remain in a region to which one ‘belongs’ and with whom one shares common values, experiences, and even turns of phrase, expressions and accents, was an important consideration in individuals’ migration decision and eventual choice of destination [54].

## 2. Data and methods

Using the individual-level census returns for 1851–1911 made available through the I-CeM project, this article matches individuals’ stated birthplaces to a GIS of nineteenth-century parishes [55–57]. Once individuals are matched to their parish of residence in each census year, their migration decision can be reconstructed [58]. Although some have argued against the use of the nineteenth-century censuses to analyse migration, suggesting that such ‘lifetime’ migration conceals intervening moves made between birth and census night, recent research has demonstrated that the transition from an individuals’ place of birth to their place of residence likely *did* take place in a single move [3, 16]. As census enumerators recorded the administrative units in which individuals were resident on census night, it is relatively straightforward to geolocate these places in a GIS. However, geolocating individuals’ self-reported birthplace is more complex, as the General Register Office (GRO) lacked the resources to standardise the responses, so much so, that the chief clerk even considered scrapping the question altogether in 1911 [59]. The task to standardise individuals’ stated birthplaces is therefore briefly outlined here.

Firstly, birthplace information in the original manuscript census returns was transcribed in the form PARISH / TOWN | COUNTY | COUNTRY, with each field being completed with as much—or as little—information as was originally recorded. Geolocating these strings was complicated by ambiguities in the birthplace strings; chiefly misspellings or non-existent places, such as ABBOTS LANGLEY | STAFFORDSHIRE | [BLANK] or strings that referred to multiple places, like NEWTON | CAMBRIDGESHIRE | [BLANK]. While the causes and consequences of such errors has been addressed elsewhere [59], the geolocating algorithm assumes that individuals made an honest attempt to describe their birthplace accurately. Therefore, unlike ‘fuzzy’ matching using Levenshtein Distance—which makes the fewest possible edits to a string in order to match it to a known place [60]–this method does not assume spelling mistakes were made (although the gazetteer incorporated as many known alternate spellings of place names as was practicable). Consequently, whereas Levenshtein Distance incorrectly matches KINGS X | MIDDLESEX | [BLANK] to Kingsland, Middlesex, this method—which assumes that the “KINGS” in “KINGS X” was intended to be a separate word—correctly matches the string to Kings Cross, Middlesex.

The algorithm which geolocated birthplace strings was therefore designed find the shortest known place name that matched the most characters in each word in the PARISH / TOWN part of the birthplace string, with the fewest redundant characters. For example, S PANCRAS | LONDON | [BLANK] matches to both Pancras, Devon and St Pancras, London on seven characters, ‘PANCRAS’, with one redundant character, ‘S’. As ‘Pancras’ is shorter than ‘St Pancras’, the first parse of the algorithm matches the string to Pancras, Devon. In pre-processing, all strings were matched to a standardised version of the county as stated in the birthplace string. As the ‘stated’ county for the string S PANCRAS | LONDON | [BLANK] was London, a second parse searches for a match in both London and the counties adjacent to London. The condition of matching to the shortest place name is removed in the second parse, so although S PANCRAS matches to both Pancras, Devon and St Pancras, London on seven characters with one redundant character, St Pancras, London is closer to the ‘stated’ county than Pancras, Devon. The second parse therefore rejects the match to Pancras, Devon and reallocates the birthplace string to St Pancras, London. The results are then checked against those produced using Levenshtein Distance [60]. Where the places to which the methods matched a birthplace string to are incongruent, precedence is given to the place that is the shortest distance from the county as stated.

Having matched all those individuals for whom a place of birth and place of residence had been specified to the GIS, an origin-destination (OD) matrix was produced. In order to identify regions of human interaction across the nineteenth-century, this article employs the COMBO algorithm which can comfortably partition a network consisting of up to 30,000 nodes [45]. Although the COMBO algorithm was chosen as it performed better than most competing community-detection algorithms, producing higher modularity scores and clearer geographical community separation, it is important to note that the differences between competing algorithms were slight and that the choice of algorithm did not significantly alter either the communities detected, or the interpretation and conclusions drawn.

Although individuals in this dataset have been matched to one of ~15,000 parishes in this dataset, such a large OD matrix would not only have been too unwieldy on which to run a SIM and evaluate the effect of these regions on migration decisions, but the unstable rates produced by small parishes would introduce too much noise, and obscure, rather than reveal, meaningful breaks in the network of human interaction [61].

Individuals’ places of birth and residence in each census year was therefore matched to one of the 2,110 registration sub-districts (RSDs) that existed in 1891. Not only does mapping census data to any consistent set of areal units aid comparison over time but the1891 census followed the 1882 Divided Parishes and Poor Law Amendment Act, rationalising British administrative geography to a greater extent than the preceding 1876 or 1879 Acts. As this programme of rationalisation continued with the 1901 and 1911 censuses—the total number of RSDs fell to 2,009 by 1911 –the 1891 RSDs are a compromise between administrative units that are large enough to minimise rate instability caused by small samples, but small enough to retain a high level of spatial resolution.

The data was adjusted further by removing individuals that had not yet left the parental home. If one is to analyse migration in a meaningful way, it is necessary to identify those that had the *capacity to migrate independently*. Attempting to infer the determinants of migrants’ ‘decisions’ would be spurious if the dataset included those for whom the decision had not have been theirs to make. Consequently, to preserve the principle that one observation represents one migration decision, only those that had left home are included. The method by which an individual is defined as having ‘left home’ is detailed in [62]. It was also supposed that the dataset should include only those for whom it could be reasonably assumed had migrated only shortly prior to census night; those migrants that had both left home and were aged within two years of the singulate mean age at leaving home (SMAL) in their RSD of birth [16]. This would ensure that the migrants sampled from one census would not be included in the sample of another, and the ‘cultural provinces’ produced from applying the community-detection COMBO algorithm to the migration flows taken from each census year, would thus be independent of one another. Any resulting similarities between the regions produced from the data in each census year could therefore be more convincingly attributed to long-standing cultural norms, rather than because observations were duplicated across samples. However, limiting the sample to just those that had not only left home, but also their RSD of birth and were also aged within two years of the SMAL, introduced small-number problems such that it could not be easily determined whether the regions produced were primarily a product of data signal, or noise.

**Fig 2:**
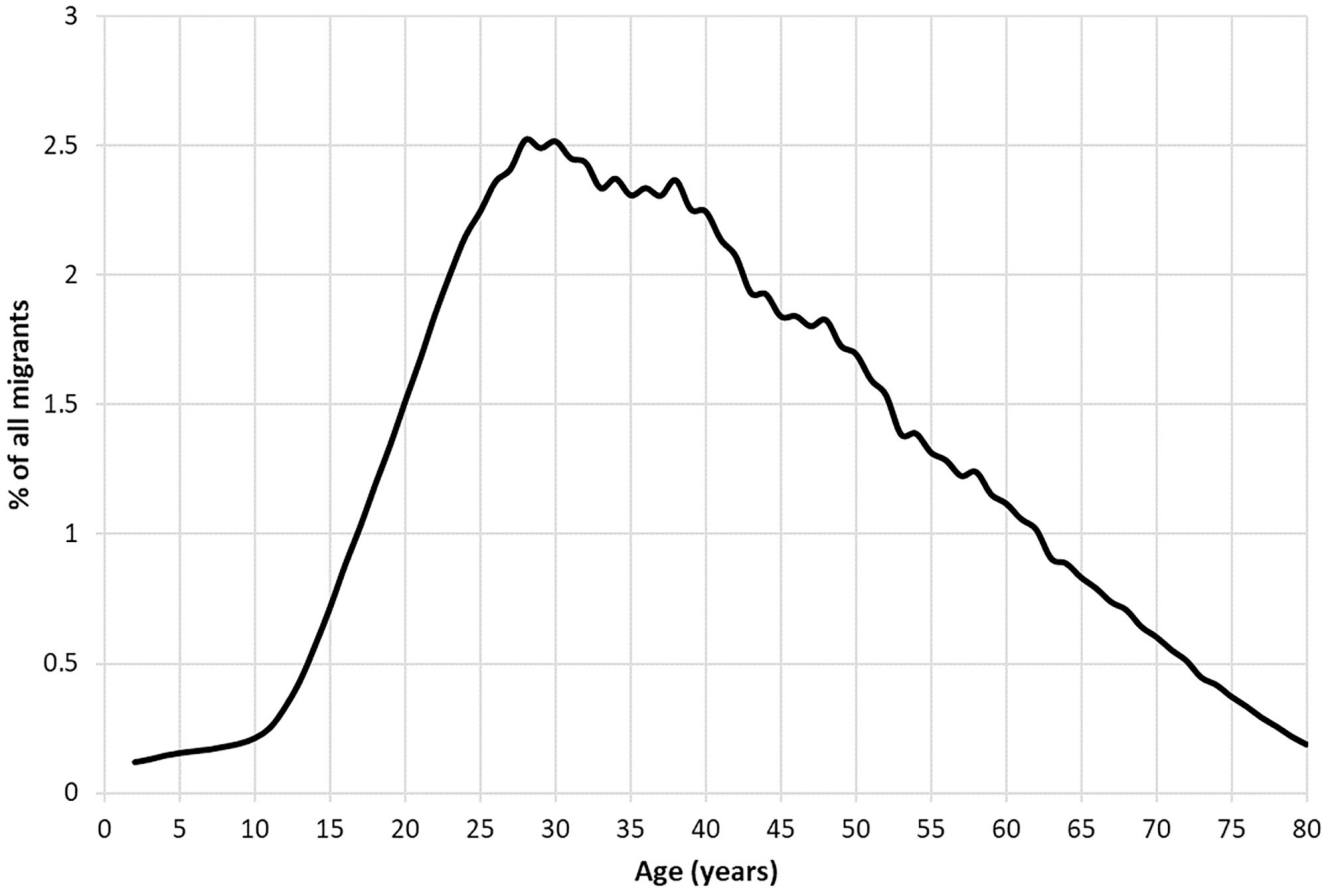
Age structure of migrants (excluding co-resident children). England & Wales 1851–1911. Source: Author’s analysis based on data from UK Data Service SN 7481 (Schürer, 2019).

In order to avoid such small-number problems, all those that had left home and moved between RSDs were included. However, as this would include *all* moves made prior to the census—rather than only those moves made shortly before census night of the year in question—the age structure of each RSD was standardised to that of all migrants in the period, as show in Fig 2 [63]. As a relatively close approximation of the age-specific moment of migration in the nineteenth century, direct age-standardisation limited the ‘smoothing’ effect that earlier waves of migration would have had on the regions produced from each census in turn [16]. While recent censuses require geographic units to be approximately the same size—output areas for example must contain no less than 40 households / 100 people and no more than 250 households / 625 people—there were no such requirements in the nineteenth century [64]. As units with larger populations would generate more connections than units with smaller populations, any potential bias was removed by standardising each RSD to a notional number of 1,000 people, thereby ensuring that the regions produced reflected purely the connectedness between RSDs. It ought to be noted that the regions produced from the full sample of migrants are not only similar to, but crucially, more robust than, the regions produced from the subset of migrants aged within two years of the SMAL. This sensitivity test means it can be confidently asserted that the regions produced represent meaningful, persistent demarcations of human interaction.

Whereas Dash Nelson and Rae [46] filtered out commutes of 262 km or more, and limited the community-detection algorithm to 50 output communities, it was decided—after much experimentation—not to add any such constraints here. Given the relative size and population density of the USA compared to England and Wales, filtering out long-distance migrants had little effect on the communities produced. Indeed, only 13.4% of the population migrated more than 100 km between 1851 and 1911, 5.1% migrated over 200 km, and just 3.3% migrated over 250 km. Limiting the number of communities produced by the community-detection algorithm was similarly unnecessary as the community-detection algorithm consistently produced between 9 and 11 regions without constraints. The next section therefore analyses the migration paths that occurred in England and Wales between 1851 and 1911 and the stability of the regions within which these moves occurred. Using these regions, it is possible to demonstrate that the pace of British urbanisation was slowed by migrants’ reluctance to move outside their ‘cultural sphere’ of origin.

## 3. Identifying the barriers to migration

**Fig 3:**
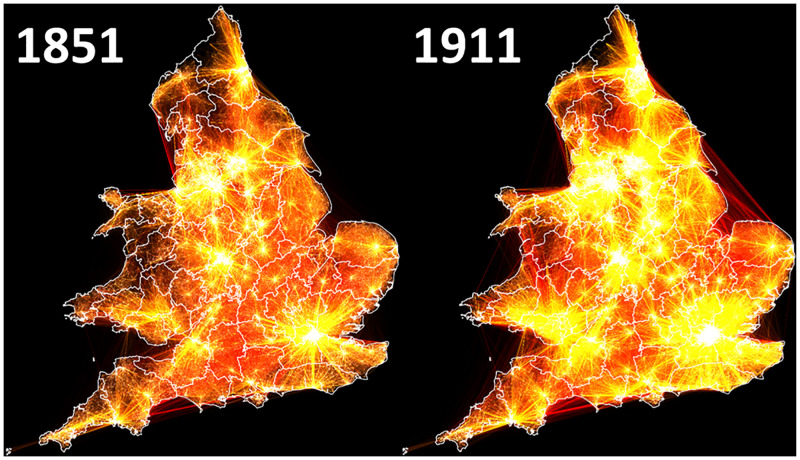
Principal migration routes. England & Wales 1851–1911. Source: Author’s analysis based on data from UK Data Service SN 7481 (Schürer, 2019).

Fig 3 illustrates the principal migration routes in 1851 and 1911 respectively and includes only those that had both left the parental home and migrated between RSDs. To aid interpretation, each line represents a single migrant, so the brighter the line, the more migrants made that move. To aid visualisation, only moves which at least 0.5% of migrants from each RSD travelled, to are included. This equated to 3,457,131 unique journeys– 72.0% of the total—in 1851, and 7,221,337 unique journeys– 60.2% of the total—in 1911. However, although Fig 3 is visually striking, it does not help clarify either the migration decisions which were dominant, or their effect on the speed of urbanisation in nineteenth-century England and Wales.

**Fig 4:**
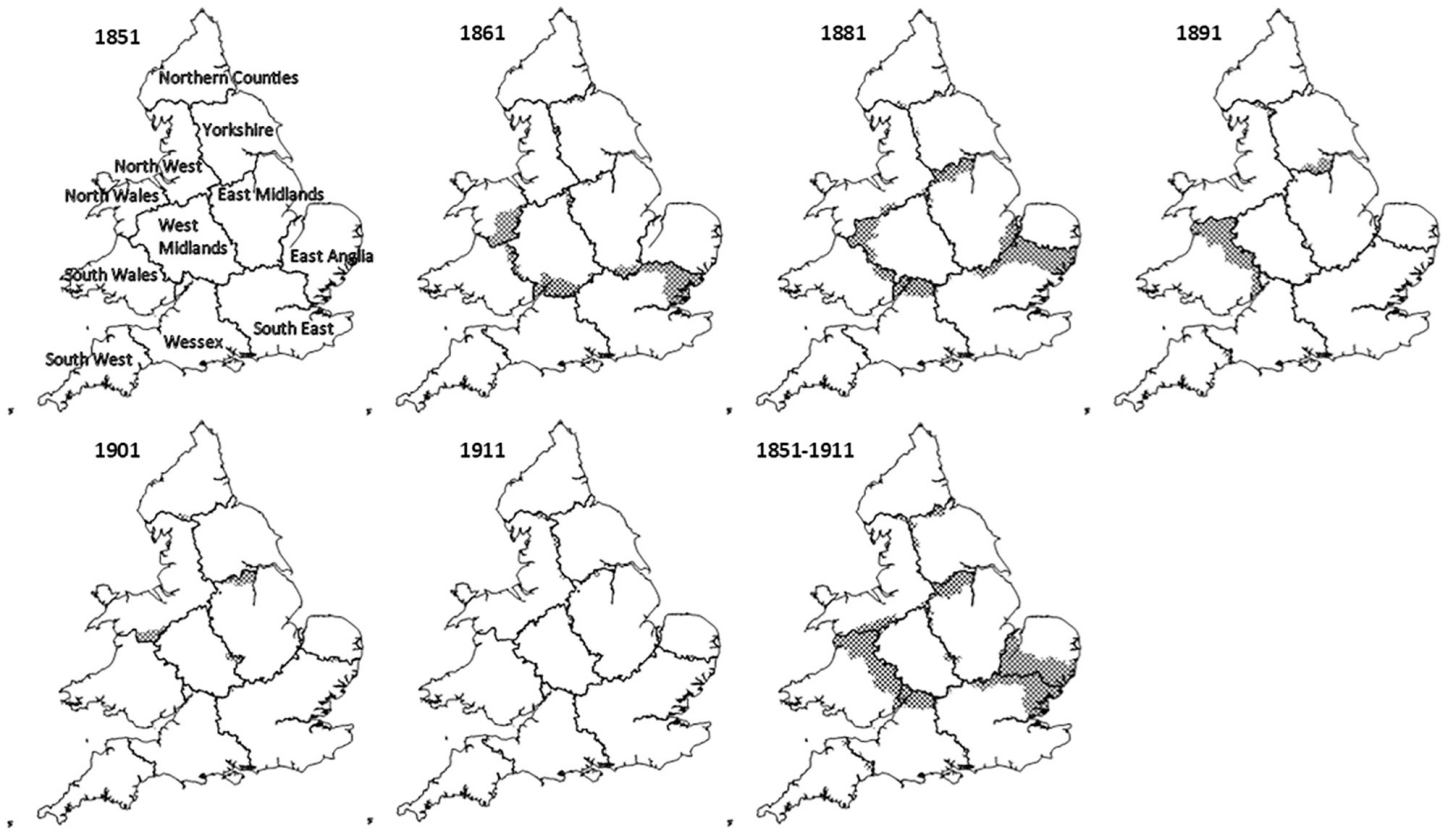
Regions produced by partitioning the migration network of England and Wales, with changes since the previous census highlighted. 1851–1911. Source: Author’s analysis based on data from UK Data Service SN 7481 (Schürer, 2019). Note: 1881 shows changes to the regions since 1861, as the 1871 census is unavailable.

The community-detection algorithm was then used to ‘partition’ the migration data and identify the principal regions within which human interaction occurred in nineteenth-century England and Wales. Fig 4 shows the regions of human interaction produced by the community-detection algorithm between 1851 and 1911. Again, it is worth reiterating that the community-detection algorithm does not include any spatial information, and that how the ‘nodes’ were grouped depended only on the volume of interaction between them.

To illustrate the relative stability of these regions over time, each panel in Fig 4 outlines the boundary of each ‘cultural provinces’ by census year, with the RSDs that were in a different cultural province in the previous census, marked by crosshatchings. The last panel shows the regions constructed from all moves between 1851 and 1911; the crosshatched areas showing those RSDs that had *ever* moved between regions.

While the relative stability of these regions over a sixty-year period is remarkable, several features deserve further scrutiny. Firstly, the volume of migration between North Wales and the North-West had increased so dramatically by 1881, that they effectively became a single region. Indeed, North Wales was so connected with the North-West, that the National Eisteddfod of Wales was held in Liverpool three times; in 1884, 1900 and 1929 –the last year the event was held outside Wales—while neighbouring Birkenhead hosted the event twice, in 1878 and 1917 [65]. To put the significance of North-West England—and Liverpool specifically—to North Wales into context, Liverpool has been dubbed the unofficial ‘capital of North Wales’, while Cardiff had only hosted the National Eisteddfod twice by 1929, once in 1883 and again in 1899 [66].

Also of note is the extent to which Herefordshire, Radnorshire and Montgomeryshire became more connected to south Wales than to the West Midlands by 1911 –likely driven by demand for labour in the coalfields—whereas the area of south Yorkshire surrounding Sheffield became more connected to the East Midlands than to the rest of Yorkshire by 1881. However, most extraordinary was the almost wholesale integration of Suffolk, Essex and Cambridgeshire—west of the River Nene—with the South East cultural province, at the expense of East Anglia, the rapidly expanding railway network likely facilitating the flight of rural labour to London during the agricultural depression [67, 68].

Other than these changes, the relative consistency of these regions over time is immediately apparent. Not only are the same regions largely recognisable over time, with most changes occurring only at the peripheries of each zone, but just 243 of the 2,110 RSDs moved between provinces and of these, 172 changed just once. The boundaries produced by partitioning the full migration network between 1851 and 1911 in the final panel—with all years equally weighted—should therefore be interpreted as the average location of the boundaries separating cultural provinces.

**Fig 5:**
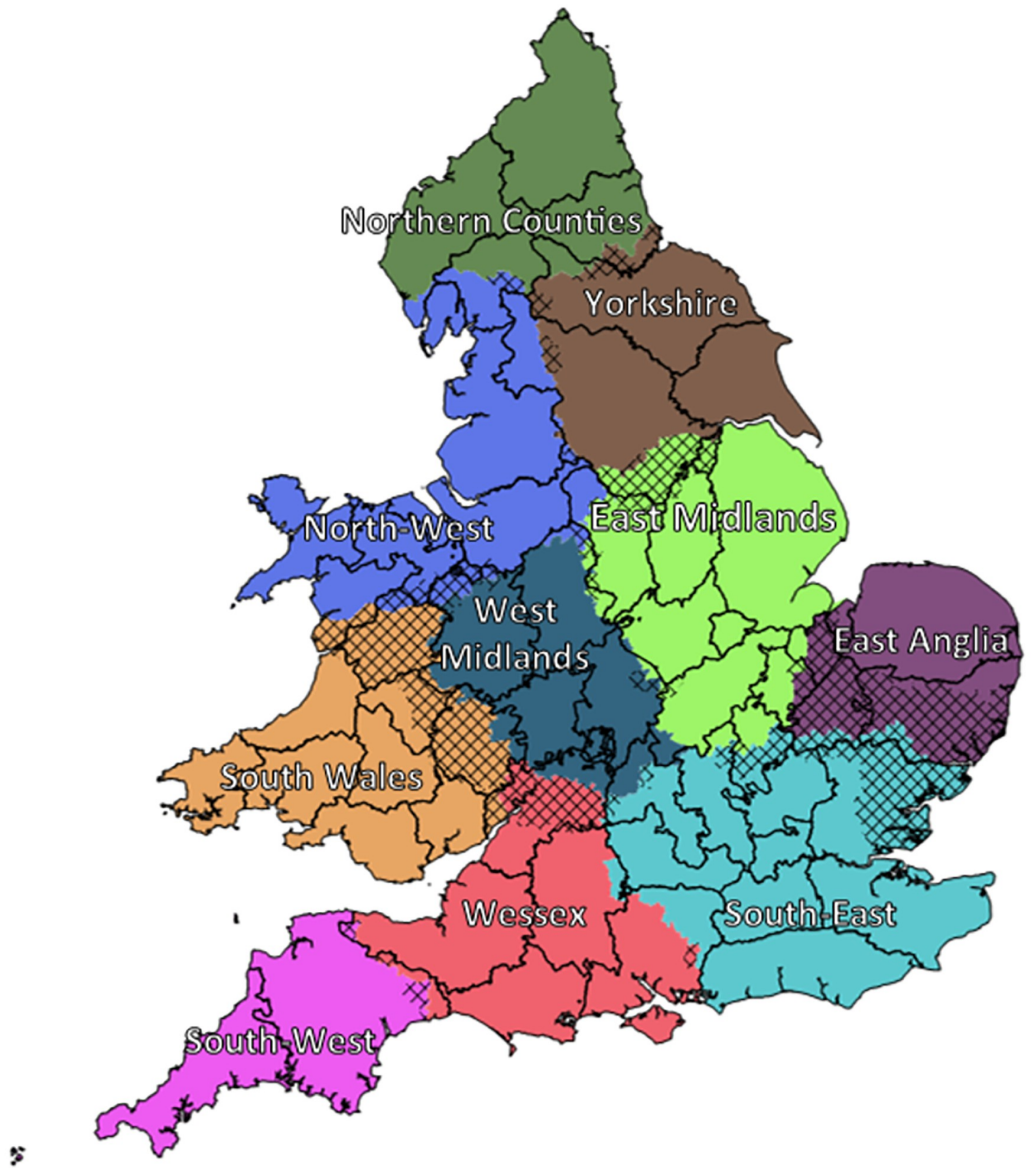
Cultural provinces of England and Wales. 1851–1911. Source: Author’s analysis based on data from UK Data Service SN 7481 (Schürer, 2019).

Overlaying the regions produced from the full network of migration between 1851 and 1911 with a map of historic county boundaries in Fig 5, it is of note that the ‘cultural provinces’ correspond well with several recognisable—and well-known—regions of England and Wales. For example, the ‘Yorkshire’ cultural province covers most of the historic North, East and West Ridings of Yorkshire, while many would consider East Anglia to be made up of Norfolk, Suffolk and Cambridgeshire [35, 40–44, 54, 69–71]. That the regions produced are so readily identifiable and easily named after long-standing and well-known regions, not only suggests that such regions are meaningful divisions of England and Wales, but also the significance of the county as an economic, political and cultural unit. Although Baines’ asserted that ‘count[y]…boundaries divided centres of population in a quite arbitrary way’, that the edges of so many cultural provinces fall either on, or close to, the borders of historic counties, seems to contradict this, and that far from being arbitrary, counties—and the boundaries between them—appear to usefully demarcate the zones of human interaction [72]. Indeed, the boundary separating the cultural province of Wessex from that of the South-West, remains virtually unchanged throughout the period and closely follows the border that Devon shares with Somerset and Dorset.

**Fig 6:**
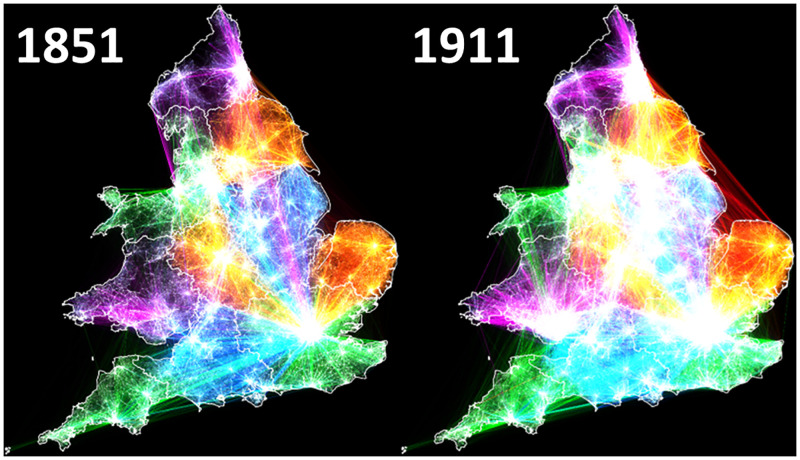
Principal migration routes, differentiated by cultural province of origin. England & Wales 1851–1911. Source: Author’s analysis based on data from UK Data Service SN 7481 (Schürer, 2019).

Therefore, whereas Fig 3 is visually striking but of little analytical merit, Fig 6 –which shows the same migration paths as Fig 3 but coloured according to their cultural province of origin—is both visually striking and analytically useful. While a cursory inspection shows that the majority of migrants did indeed remain in their region of origin– 76.5% in 1851 –the increase in long-distance migration over the period reduces this to 71.5% by 1911. Most striking is the importance of London in the period; drawing in migrants from across England and Wales, as well as the growth of the Glamorganshire coalfields by 1911; drawing in former copper miners from North Wales, while migrants from Norfolk became increasingly likely to cross the Wash, migrating to both Hull, and beyond to Middlesbrough [73].

This increase in the proportion of migrants crossing the boundaries of cultural provinces between 1851 and 1911 is reflected in the modularity score. As a measure of community structure, the modularity scores of the ‘optimal’ regions produced by partitioning the migration networks between 1851 and 1911 –as shown in Fig 4 –declined from 0.657 in 1851 to 0.628 in 1861, 0.601 in 1881, 0.583 in 1891, 0.576 in 1901 and finally to 0.567 by 1911, with a modularity score of 0.596 when partitioning the full 1851–1911 network. That these regions became less internally cohesive over time—with more migrants moving between cultural provinces—could be interpreted as evidence that while such boundaries might be an ‘optimal’ division in the network of nineteenth-century human interaction, they are not necessarily statistically significant and may instead represent arbitrary—and therefore meaningless—regions of human interaction [74, 75].

To demonstrate that the boundaries separating the cultural provinces were significant, meaningful barriers to migration throughout the period 1851–1911 –despite having become more permeable by the dawn of the railway age—it would be instructive to analyse the impact which these boundaries had on limiting the volume of migrants which crossed them. The next section therefore analyses the effect which boundaries had on migration, by formally including whether such a boundary had been crossed or not, into a spatial interaction model (SIM) as an independent dummy variable. By analysing the effect which boundaries had on rural to urban migration specifically, their impact on the pace of British urbanisation can be seen and quantified.

## 4. Modelling the barriers to migration

A SIM was therefore produced to quantify the extent to which such imagined boundaries represented real and meaningful barriers to both migration and the speed of urbanisation. Although a comprehensive overview of SIMs can be found in [14, 76, 77], broadly speaking, SIMs have their origins in Newtonian physics and the law of universal gravitation that ‘any two bodies attract one another with a force that is proportional to the product of their masses [population] and inversely proportional to the square of the distance between them’ [78]. Following Zipf [10], this can be specified in Eq 1 below as:

$$M_{ij}=k\frac{P_{i}P_{j}}{d_{ij}^{2}}$$ (1)

Where *M*_*ij*_ is the flow of migrants *M* between origin *i* and destination *j*, *P*_*i*_ is the population *P* at origin *i*, *P*_*j*_ is the population *P* at destination *j* and $d_{ij}^{2}$ is the square of the distance *d* between origin *i* and destination *j*. *k* is the constant of proportionality [10, 79–81]. The model can then be respecified as a Poisson loglinear regression model in Eq 2 below [82, 83].

$$\lambda_{ij}=exp\left({\beta_{1}ln{\;P}_{i}+\beta }_{2}ln{\;P}_{j}+\beta_{3}ln{\;d}_{ij}\right)$$ (2)

Where λ_*ij*_ is the conditional mean of the predicted migration flow between origin *i* and destination *j*. The parameters *β* are estimated by the model and show the effect of each variable on the volume of migration between any two given locations.

In Wilson’s ‘family’ of spatial interaction models, he presented the origin-constrained model—in which all predicted migrant flows sum to the observed number of migrants from each origin—the destination-constrained model—in which all predicted migrant flows sum to the observed number of migrants at each destination—the doubly-constrained model—in which all predicted migrant flows sum to the observed number of migrants from each origin *and* at each destination, and the unconstrained model. The number of predicted migrant flows in an unconstrained model must only sum to the *total* observed migration flows and it may therefore predict greater or fewer migrant flows to/from each destination/origin than are observed [8]. An origin-constrained model was thought to be the most appropriate, as in reality, the number of migrants at an origin is similarly constrained to the numbers observed, while the number of migrants at a destination is determined by the relative push and pull factors of the origin and competing destinations. Consequently, an origin-constrained model includes *μi* as a fixed ‘origin’ effect and is akin to the balancing factor *A*_*i*_ in Wilson’s original Entropy Maximising Model [8]. In other words, each origin is included in the model as a separate dummy variable, ensuring the number of migrants at each origin predicted by the model, equals the number observed. However, if each origin is represented as a separate dummy variable, this would make *P*_*i*_—and other origin-specific predictors—redundant [84, 85] and thereby endogenize many of the ‘push’ determinants of migration which it would be desirable to explicate in the model. The model presented is therefore unconstrained [86]. By respecifying the ‘gravity model’ in Eq 1 as a regression model in Eq 2, further explanatory variables beyond just population and distance can be added to the model. Although these are listed in full in Table 1, it would first be useful to outline their provenance, interpretation, and relevance to migration.

**Table 1:** Determinants of migration between origin *i* and destination *j*. England and Wales, 1851–1911.

				1851	1861	1881	1891	1901	1911	1851–1911
**Origin-specific parameters**	Settlement *T*_*i*_	Outside London		-9.17	-25.02	-21.07	-23.46	-12.54	-13.98	-27.90
		Inside London		-9.93	-25.55	-21.70	-24.23	-13.38	-14.68	-28.80
	Industry *I*_*i*_	Agriculture		*(ref*.*)*	*(ref*.*)*	*(ref*.*)*	*(ref*.*)*	*(ref*.*)*	*(ref*.*)*	*(ref*.*)*
		Semi-Rural		-0.18	-0.18	-0.21	-0.38	-0.49	-0.33	-0.10
		Mining		-0.15	-0.22	-0.31	-0.31	-0.28	-0.34	-0.09
		Textiles		-0.48	-0.53	-0.32	-0.46	-0.54	-0.60	-0.17
		Urban (Other)		0.02	-0.29	-0.30	-0.37	-0.22	-0.43	-0.12
		Professional		0.12	-0.44	-0.34	-0.50	-0.20	-0.08	-0.13
		Semi-Professional		0.08	-0.37	-0.19	-0.39	-0.17	-0.18	-0.11
		Transport		0.16	-0.68	-0.33	-0.24	-0.19	-0.31	-0.21
	% of non-migrants	ln (*M*_*i*_)		-0.68	-0.60	-0.88	-0.59	-0.27	-0.19	-0.98
	% of non-migrants x Area	ln (*M*_*i*_) × ln (*A*_*i*_)		0.15	-0.06	-0.03	0.00*	-0.02	-0.06	-0.06
	Population	ln (*P*_*i*_)		0.85	1.97	1.93	1.62	1.32	1.57	2.19
	Population density	ln (*D*_*i*_)		0.17	-0.44	-0.34	-0.20	-0.27	-0.39	-0.40
	Population change (lag)	ln (*P*_*i*(*x*−10)_/*P*_*i*(*x*−20)_)		-1.52	-0.17	-0.20	-0.30	-0.96	-0.90	-0.48
	HiS-CAM score	ln (*H*_*i*_)		0.20	1.08	0.47	2.26	1.54	0.39	1.59
	ln(PageRank)	ln (*R*_*i*_)		-0.54	-1.08	-1.26	-1.08	-0.66	-0.81	-1.22
**Destination-specific parameters**	Settlement *T*_*j*_	Outside London		*(ref*.*)*	*(ref*.*)*	*(ref*.*)*	*(ref*.*)*	*(ref*.*)*	*(ref*.*)*	*(ref*.*)*
		Inside London		-0.38	-0.27	-0.30	-0.36	-0.36	-0.37	-0.32
	Industry *I*_*j*_	Agriculture		*(ref*.*)*	*(ref*.*)*	*(ref*.*)*	*(ref*.*)*	*(ref*.*)*	*(ref*.*)*	*(ref*.*)*
		Semi-Rural		-0.08	-0.06	-0.08	-0.05	-0.12	-0.09	0.01
		Mining		0.07	0.03	0.10	0.08	-0.01*	0.06	0.07
		Textiles		-0.31	-0.42	-0.28	-0.26	-0.29	-0.24	-0.21
		Urban (Other)		-0.04	-0.21	-0.11	-0.10	-0.10	-0.14	-0.03
		Professional		-0.04	-0.12	0.02	-0.01	0.07	0.03	0.06
		Semi-Professional		0.00*	-0.20	-0.05	-0.04	-0.01	-0.10	-0.02
		Transport		-0.02	-0.36	-0.02	-0.04	0.03	-0.01	0.00
	% of non-migrants	ln (*M*_*j*_)		0.00*	-0.03	0.00	-0.04	-0.05	-0.03	0.04
	Population	ln (*P*_*j*_)		0.59	0.62	0.57	0.59	0.59	0.63	0.51
	Population density	ln (*D*_*j*_)		-0.20	-0.17	-0.19	-0.19	-0.23	-0.25	-0.19
	Population change (lag)	ln (*P*_*j*(*x*−10)_/*P*_*j*(*x*−20)_)		-0.17	0.11	-0.12	0.09	0.04	0.01	-0.01
	HiS-CAM score	ln (*H*_*j*_)		1.73	2.24	1.88	1.21	0.71	1.41	1.98
	ln(PageRank)	ln (*R*_*j*_)		0.62	0.50	0.61	0.61	0.65	0.61	0.65
**Interaction Parameters**	Origin	Destination								
	Rural	Rural		*(ref*.*)*	*(ref*.*)*	*(ref*.*)*	*(ref*.*)*	*(ref*.*)*	*(ref*.*)*	(ref.)
		Urban		-2.00	-1.87	-1.57	-1.63	-1.68	-1.52	-1.63
	Urban	Rural		-1.76	-1.80	-1.68	-1.73	-1.72	-1.45	-1.83
		Urban		-3.67	-3.48	-3.33	-3.18	-2.89	-2.32	-3.72
	Boundary not crossed			*(ref*.*)*	*(ref*.*)*	*(ref*.*)*	*(ref*.*)*	*(ref*.*)*	*(ref*.*)*	*(ref*.*)*
	Boundary crossed *Z*_*ij*_	Origin	Destination							
		Rural	Rural	-0.56	-0.78	-1.04	-1.19	-1.23	-1.55	-1.11
			Urban	-0.48	-0.77	-1.03	-1.23	-1.30	-1.66	-1.12
		Urban	Rural	-0.48	-0.76	-1.06	-1.23	-1.31	-1.61	-1.15
			Urban	-0.56	-0.81	-1.21	-1.29	-1.22	-1.50	-1.26
	Distance migrated ln *d*_*ij*_	Origin	Destination							
		Rural	Rural	-2.34	-2.20	-2.19	-2.17	-2.16	-2.09	-2.27
			Urban	-1.70	-1.63	-1.72	-1.69	-1.67	-1.64	-1.78
		Urban	Rural	-1.75	-1.67	-1.69	-1.68	-1.70	-1.70	-1.81
			Urban	-1.03	-1.05	-1.10	-1.18	-1.29	-1.38	-1.12
	Distance migrated ln *d*_*ij*_	Boundary not crossed		*(ref*.*)*	*(ref*.*)*	*(ref*.*)*	*(ref*.*)*	*(ref*.*)*	*(ref*.*)*	*(ref*.*)*
		Boundary crossed		-0.04	0.02	0.06	0.09	0.10	0.16	0.07
	Distance (origin to boundary) ln *d*_*iz*_	Boundary not crossed		-0.01	-0.04	-0.07	-0.07	-0.06	-0.11	-0.08
		Boundary crossed		0.12	0.08	0.12	0.12	0.14	0.11	0.12
Pseudo *R*^2^				0.449	0.351	0.356	0.396	0.422	0.433	0.380

Source: Author’s analysis based on data from UK Data Service SN 7481 (Schürer 2019)

Note: All variables are significant at the <0.01 confidence level. Those marked with an asterisk (*) are not significant.

First, both origin- and destination-specific parameters include a dummy variable describing the predominant industry in each RSD in each census year. The classifications used here are based on those used by www.PopulationsPast.org, which was selected over a range of competing alternatives [87, 88] for its simplicity and ease of interpretation. London is also included as a separate predictor. While urban/rural classifications are included as interaction effects—rural to urban, urban to rural etc., represented by parameter *U*_*ij*_—London is included as a main effect, separate to other urban destinations given its unique status in both the British economy and urban hierarchy [16, 88]. Indeed, as London has always been the primate British city—its size far exceeding that predicted by a rank-size distribution—the attractions of London might not be fully captured by the other explanatory variables in the model and instead, London’s more intangible—and perhaps unquantifiable—attractions might be better captured by a dummy variable [89, 90].

While population and population density in origin *i* and destination *j* are relatively self-explanatory, population change is incorporated as a lagged variable, represented by the expression (*P*_*i*(*x*−10)_/*P*_*i*(*x*−20)_) where *x* is the census year. While population growth is a good proxy for the attractiveness of a destination and may endogenize factors not adequately captured by other predictors, it is itself a product of the process being modelled—migration—and hence to use population growth to predict migration which is a cause of population growth, introduces circularity. A lagged variable—where migration in 1881 is predicted by the population growth between 1861 and 1871, thereby captures the growth and popularity of a destination in 1881, without being a component of migration in 1881.

The age-standardised proportion of non-migrants is also included as predictors for both the place of origin and destination, as an additional measure of a location’s attractiveness, assuming that few people leave places that attract migrants. However, as one would expect larger RSDs to have fewer out-migrants than smaller RSDs, the proportion of non-migrants is partly dependent on the size of the RSD. The model therefore includes the acreage of migrant’s RSD of origin as an interaction effect with the proportion of non-migrants. The age-standardised average HiS-CAM score of both origin *i* and destination *j* is also included as a measure of average skill level of available work and therefore wages, as a further estimate of the attractiveness of competing destinations [60, 91–95]. HiS-CAM scores measure social interaction and as such, are used to stratify occupations from the highest to the lowest social class. HiS-CAM should therefore be interpreted as a measure of occupational skill. In the absence of high-quality wage data and, given that wages are—in part—a function of skill, HiS-CAM scores are taken as a very rough proxy of wages.

Briefly, HiS-CAM is a historical version of the CAMSIS approach to determining the relative stratification position of incumbents of various occupations based on their level of interaction with individuals in other occupations. The CAMSIS measures are constructed using data on pairs of occupations linked by a social interaction such as marriage, friendship, or parent-child relationship. By asking, for example, how many friends of bakers are bakers/butchers etc., the relative social distance between these occupations is established. The scale was then standardised between 0 and 100, with 50 as the median. Several scales were produced, generated from the given occupations of bridegrooms’ and brides’ fathers listed in marriage registers. Versions 1.3.1.E and 1.3.1.L were produced using intergenerational occupational pairs in seven countries from 1800–1890 and 1890–1938 respectively. Version 1.3.1.E was used to measure social stratification in the 1851–1881 censuses, while version 1.3.1.L was used for the 1891–1911 censuses.

PageRank centrality is also included as a predictor of migration flows. However, as this is a more complex measure of a destinations’ attractiveness to potential migrants, it warrants a little further explanation. Fotheringham argued that spatial interaction models are likely to be mis-specified where they do not account for variations in the spatial structure, and that the destinations which ‘compete’ for migrants, will differ depending on the migrants’ origin [96–100]. While Fotheringham developed a theory of competing destinations and an ‘accessibility index’ to measure the origin-specific accessibility of each destination relative to competing ones, its calculation is based on the population and distance of such “competing destinations” to each origin; variables which are already included in the SIM. PageRank centrality scores on the other hand uses the observed network of migration to identify the destinations—or ‘nodes’–that are more/less important in the migration network [101]. Adapted from Graph Theory and Search Engine Optimisation (SEO), PageRank centrality finds the probability that from any given starting point in the network and a 0.15 chance that the next move will be their last, a migrant will end up at a particular destination RSD/node [101]. Consequently, and as noted by Charyyev and Gunes in their well-turned phrase assessing the measure: *“PageRank centrality adjusts the generous recognition of the eigenvector centrality*, *and ranks a node higher if it is linked from other important and stingy nodes or if it is highly linked”* [102]. As a measure of a node’s importance in a network, PageRank centrality can be conceptualised as an additional measure of a destination’s attractiveness, beyond those captured by its size, growth, or the nature of the local economy.

Lastly, in addition to urban/rural origins/destinations, the distance migrated and—of course—whether a boundary is crossed or not, the distance that migrants were born from a boundary, is also included as a predictor. How far a migrant was born from a boundary will likely have an effect on their likelihood of crossing it and so the interaction between the distance that migrants’ origins were from a boundary and whether it is crossed or not, is also included as an effect in the model.

Most model outputs were as expected; for example, being born inside London limited the number of out-migrants relative to those born outside, likely because both the wages and amenities offered by London made alternatives less appealing. As a destination however, London attracted fewer migrants compared to other—urban and rural—destinations. While it might be argued that the attractions of London were adequately captured by other variables in the model, it nonetheless demonstrates that London’s status as the capital city of an empire, was not a sufficient attraction to prospective migrants in and of itself.

Like London, migrants from textile RSDs were also less likely to migrate relative to migrants from elsewhere. While relatively high wages in textile towns would undoubtedly have been a factor in deterring out-migration, wages were higher still in both mining and the professions; meaning a more convincing explanation would be the relative skill and geographical distribution of textile trades compared to other industries. While professional jobs were highly skilled, there was little agglomeration of such services outside the capital, necessitating professionals to migrate for jobs that tended to be relatively diffuse [103–105]. Consequently, although migrants from RSDs dominated by the professions were less likely to leave than those from agricultural RSDs, they were more likely to do so than those from textile towns. Mining jobs on the other hand were—although obviously concentrated on coalfields—fairly unskilled, allowing miners relative freedom to move across the country, making them similar to professionals; less mobile than agricultural labourers, but more mobile than textile workers, whose work was both highly specialised and agglomerated, with the result that there was virtually no demand for a textile workers’ skill outside of the cloth industry, inhibiting out-migration from textile areas more than others [102–104]. This interpretation is compounded by the observation that as destinations, textile towns inhibited in-migrants—the skill required effective barring all but natives, whereas RSDs dominated by high-paid, low-skilled mining, consistently *attracted* migrants [106].

However, as this paper models only migration between RSDs, migrants from larger RSDs would have to travel a greater distance to count as a migrant compared to those born in smaller RSDs. Consequently, larger RSDs endogenize an unobserved distance-decay effect [107]. As a proxy for this unobserved distance-decay effect within RSDs, the area of a migrants’ RSD of birth is interacted with the proportion of its native population that did not migrate outside of it, in order to distinguish the distance-decay effect—which limits individuals’ *capacity* to leave their RSD of birth—from the intrinsic appeal of an RSD—which limits their *incentive* to do so. Consequently, using the proportion of non-migrants to proxy the innate attractions of an RSD, shows that—unsurprisingly—rates of out-migration were lower, the more popular it was with natives. However, even when the intrinsic attractiveness of an RSD is accounted for, larger RSDs limited the number of out-migrants in all census years except 1851. This highlights the importance of incorporating the unobserved distance-decay effects that occur *within* units into SIMs, as well as the observed distance-decay effect *between* units.

Also as expected, RSDs with larger populations resulted in more in-/out-migrants, while densely populated places tended to deter both in- and out-migration. It could be argued that while natives of such RSDs had little incentive to leave—it would not be densely populated were it not popular—they might also be less attractive to prospective in-migrants given that such destinations might be perceived as being ‘full’. Similarly, a place that was growing rapidly limited the number of out-migrants—given the obvious demand for labour—while population growth seems to have had a more equivocal effect on in-migrants. While one might expect that migrants would be attracted to rapidly growing places, this does not appear to have universally been the case, limiting the number of in-migrants in 1851 and 1881. More explicable however, is that places with high HiS-CAM scores increased both out-migration—as skilled workers sought better opportunities elsewhere—and attracted in-migrants, doubtless drawn from agricultural RSDs in search of higher wages [29]. As a further measure of the attractiveness of an RSD, PageRank centrality shows that migrants were not only drawn to well-connected places, but that natives were similarly reluctant to leave them.

While the aforementioned determinants of migration are of interest in their own right, in terms of this paper’s stated purpose, their relevance is primarily as controls for the boundary effect. In order to determine whether boundaries inhibited migration generally and urbanisation specifically, the interaction parameters analyse the interactions between urban/rural origins and urban/rural destinations. It appears that relative to rural-rural transfers, all other types of moves were inhibited, but likely for very different reasons. First, it is useful to view rural-rural moves as part of a complex network of circulating rural labour, with a significant number of rural-born men moving short distances to neighbouring farms to work as agricultural labourers. The move to towns and cities on the other hand represented a more permanent move, likely inhibiting the volumes of rural to urban moves relative to moves between rural places [16]. As rural places offered fewer opportunities than urban places, the majority of urban to rural migrants were limited to the relatively small group for whom an urban to rural move was upwardly mobile, such as newly ordained curates joining their first parish after leaving the seminary [108]. Moves made between urban places were similarly less likely than circulating rural moves, as, while the relative gains made by moving from the countryside to the town were significant, the gains made by moving from one town to another were less so, such that there would have been precious few reasons to migrate from one town to a similar one.

Although it would not be unreasonable to suppose that cultural boundaries represented less of a barrier to rural-urban migrants than circulating rural migrants, it appears instead that such boundaries inhibited rural-urban migration more even than rural-rural migration from 1891 onwards. While the effect of boundaries does not appear to have been as dependent on the urban/rural classification of migrants’ origin/destination as might have been supposed, the distance-decay effect certainly was. Indeed, rural-rural migrants were the most inhibited by distance, unwilling to travel far to make such a minimal gain, while urban-urban migrants were the least inhibited by distance. This may have been a consequence of good intercity rail links, compressing physical distance, or the relative homogeneity of towns and cities, compressing cultural distance [109].

It is also of note that—as might be predicted—the distance-decay effect was marginally weaker when a cultural boundary was crossed relative to when it was not, as migrants that were not impeded by cultural barriers were also less impeded by distance. Lastly, it is worth noting that the extent to which boundaries between cultural provinces represented barriers to migration is, in part, a function of how far potential migrants were from said boundary. An interaction effect between the distance that migrants had been born from a cultural boundary and whether a boundary was crossed or not, is therefore included to control for this.

Therefore, having controlled for other determinants of migration, regional boundaries had a significant, limiting effect on the volume of migrants flowing across them. That boundaries also had a clear limiting effect on the volume of rural to urban migration specifically, demonstrates that such cultural barriers did indeed slow migration and especially the pace at which Britain urbanised.

**Fig 7:**
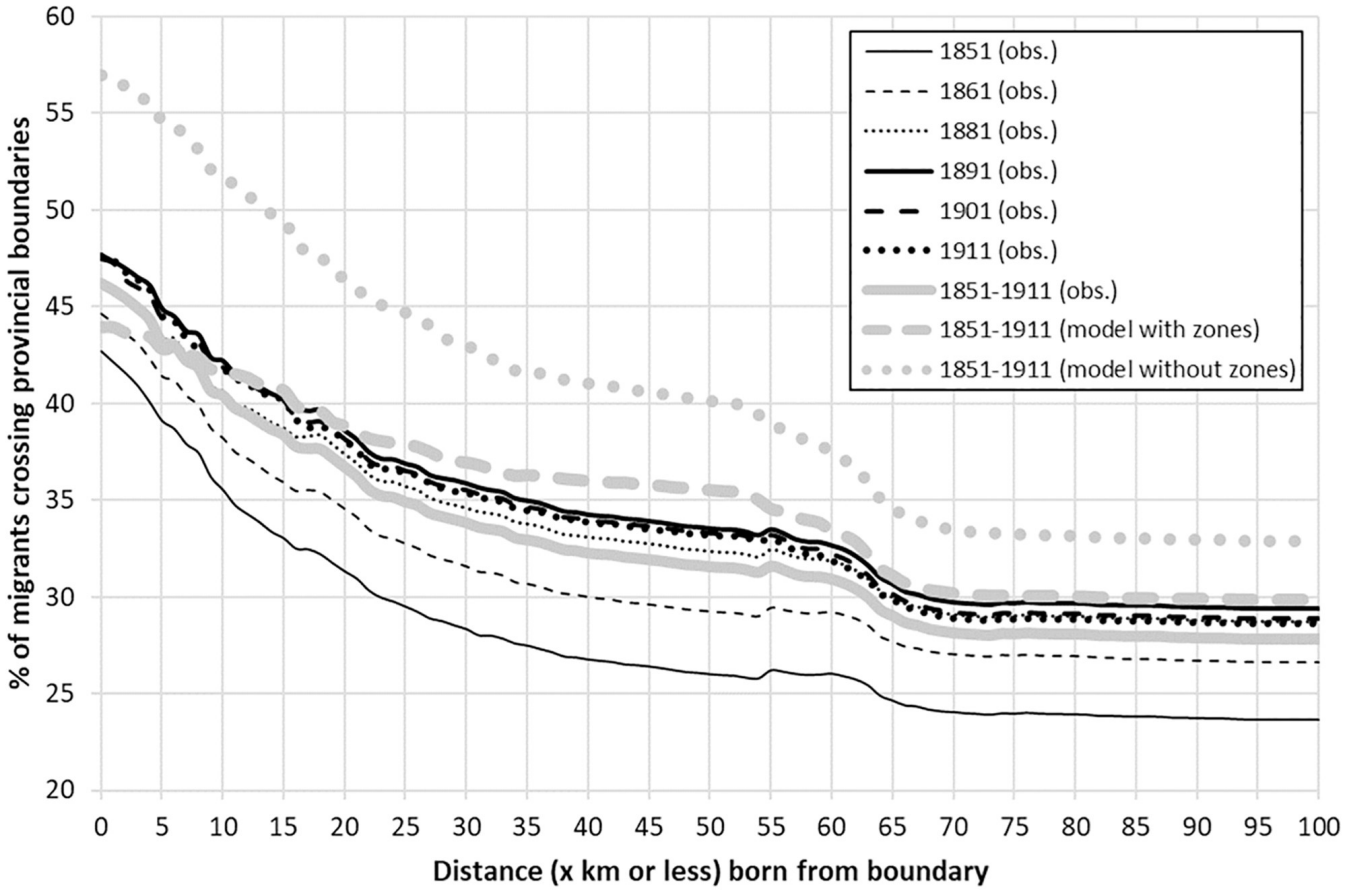
Proportion of migrants that cross a provincial boundary by those born ‘x km or less’ from such a boundary. Age-standardised. England & Wales, 1851–1911. Source: Author’s analysis based on data from UK Data Service SN 7481 (Schürer, 2019).

However, in order to better visualise the effect which regional boundaries had on individuals’ migration decision—and demonstrate that they were not arbitrary demarcations, but real and meaningful barriers to interaction–Fig 7 analyses the proportion of migrants that crossed a boundary, relative to the distance they had been born from one, and compares this to the proportions predicted to have done so by the model presented in Table 1, when boundary-specific parameters are/not included, respectively. Several features of Fig 7 are worth noting. Firstly, it is revealing that across the period, less than half of migrants born in an RSD that was *on* a provincial boundary—expressed here as being 0 km from such a boundary—crossed it. That boundaries had a clear effect on the *direction* of migration is compounded when comparing the number of migrants predicted to have crossed a cultural threshold when such boundaries are not included as predictors.

Using all migration flows between 1851 and 1911, the data series labelled ‘1851–1911 (model without zones)’ shows that when the interaction terms which include the variable *Z*_*ij*_—the boundary effect—are excluded from the model presented in Table 1, we would expect that *ceteris paribus*, 56.98% of migrants living on a border would have crossed it, whereas the full model as presented in Table 1 which includes all *Z*_*ij*_ ‘boundary effect’ interaction terms, estimates that just 43.95% would do so. This is far closer to the observed 46.23%, suggesting that such ‘cultural’ boundaries do indeed also act like barriers. Indeed, Fig 7 shows that such barriers lowered the proportion of migrants that moved between cultural provinces by approximately 10–20%, regardless of the distance they were born from such a boundary. Indeed, that the inclusion of boundaries in the model so accurately predicts the proportion of migrants that crossed them relative to when they are not included, demonstrates their significance as determinants, not only of the volume of migration, but its *direction*.

Although Table 1 –and by extension Fig 7 –indicates that the boundaries separating each province significantly limited the number of migrants moving between them, such a conclusion would lack validity if the standard assumptions of regression modelling were not met. While the explanatory variables lacked multicollinearity, displayed a linear relationship with the dependent variable, and the residuals appeared to have been normally distributed and homoscedastic, the errors did not appear to be independent, but rather, exhibited spatial autocorrelation [110]. In other words, under/overestimates tend to near one another, violating the assumption that all observations are equally likely to contain errors of a similar magnitude [111].

**Fig 8:**
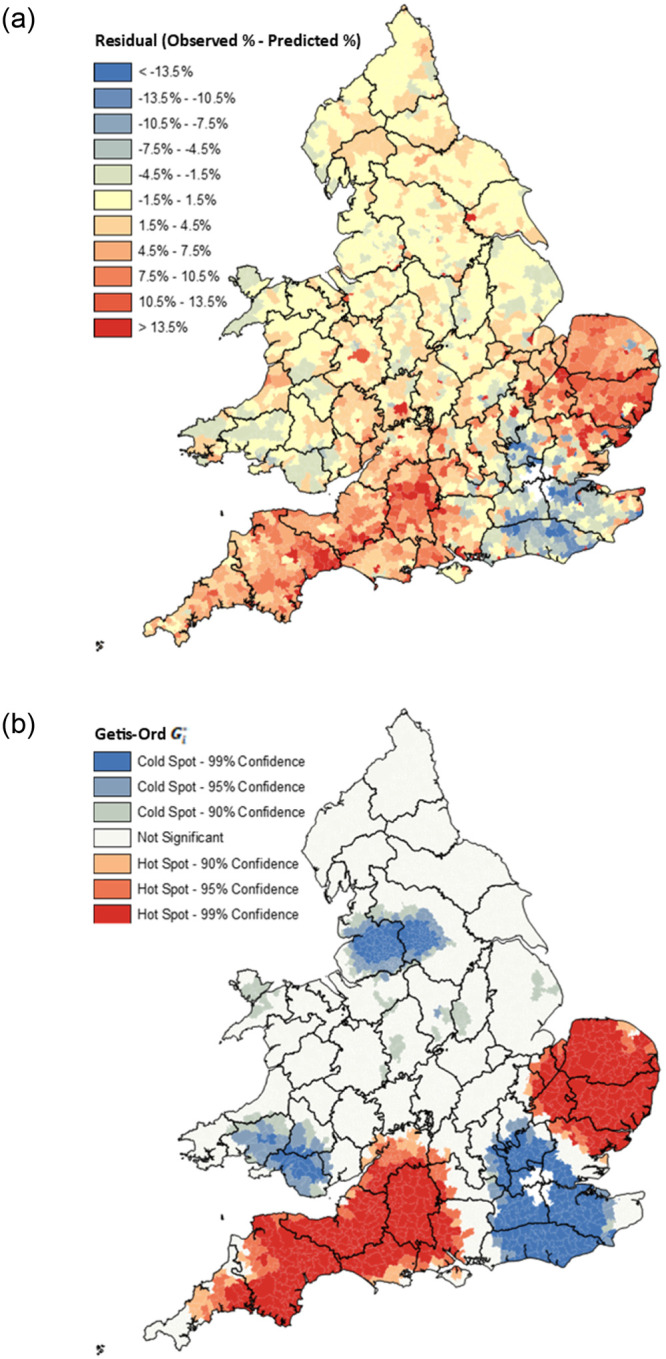
Residuals between observed age-standardised proportion of London-bound migrants from each RSD and that predicted by model from Table 1. England & Wales 1851–1911. **a) Raw Residual b) Residual Hot/Cold Spots.** Source: Author’s analysis based on data from UK Data Service SN 7481 (Schürer, 2019). Note: London is defined by its 1911 boundaries [16, 88].

To illustrate this, Fig 8 maps the residual between the proportion of the migrant population observed to have migrated to London and the proportion predicted to have done so by the model presented in Table 1 between 1851 and 1911. Panel a) maps the raw residual, and this shows extremely clearly that the model predicts the number of migrants born north of the Wash-Severn line that headed to London tolerably well, whereas far more migrants travelled to London from East Anglia and the South-West than predicted, whereas far fewer did so from the South East.

**Fig 9:**
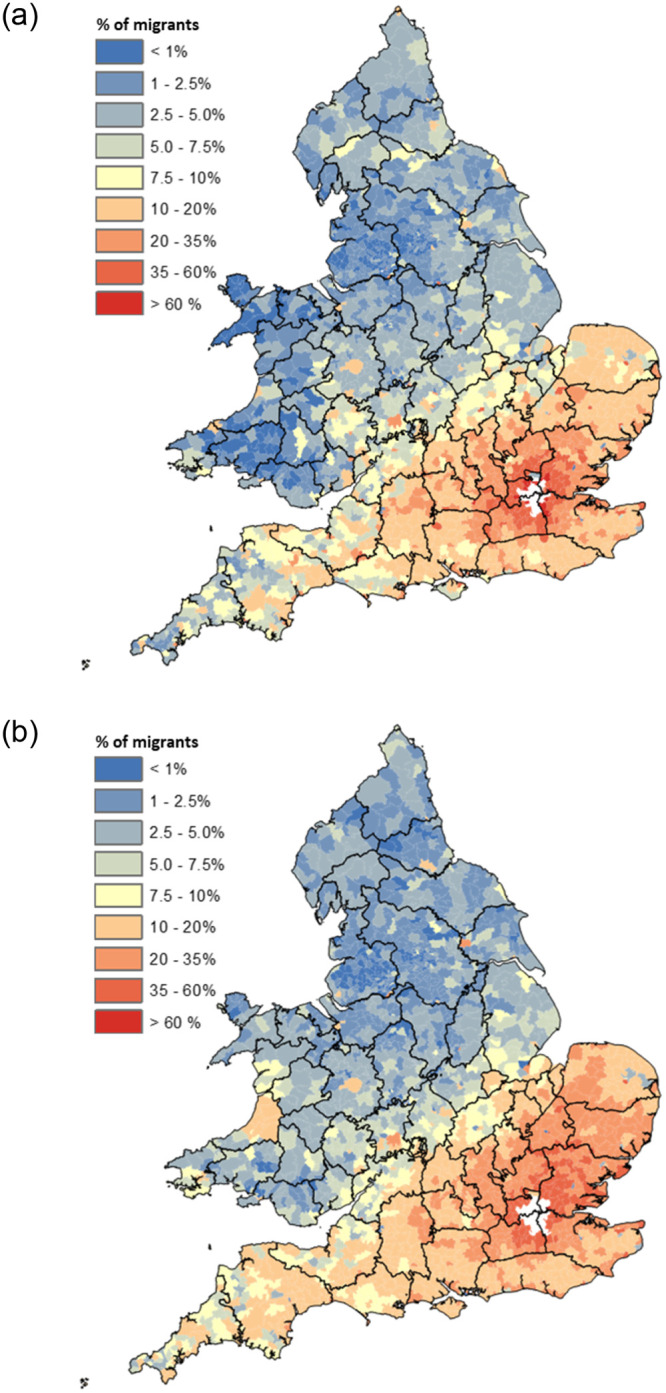
Age-standardised proportion of London-bound migrants from each RSD. England & Wales 1851–1911. **a) 1851, b) 1911**. Source: Author’s analysis based on data from UK Data Service SN 7481 (Schürer, 2019). Note: London is defined by its 1851 boundaries in panel a) and by its 1911 boundaries in panel b) [16, 88].

The importance of this observation is exemplified in panel b) which shows statistically significant clusters of high/low residuals, calculated using the Getis-Ord $G_{i}^{*}$ [112, 113]. This reiterates the observation made in panel a) that migrants to London were far more likely to come from East Anglia and the South-West than is predicted by the model [114]. While this ought to be of little surprise given the numbers observed to have migrated to London from East Anglia and the South-West—as shown in Fig 9 –spatially autocorrelated residuals violate the standard assumptions in regression analysis which dictate that residuals ought to be random and therefore independent of one another, biasing the model [111]. This is because the model described in Table 1 is a *global* model, and therefore assumes that the relationships between the dependent variable and each of the independent variables are invariant over space.

Yet as Fotheringham and Pitts noted, it is a little odd that geographers seek to explain spatial variation in the dependent variable by assuming that its relationships *between* with the independent variables is spatially invariant [53]. As it is likely that migrants from one part of England or Wales had a greater preference for a destination compared to migrants from elsewhere—for reasons not captured by the model presented in Table 1 –it is also likely that migrants from different parts of England and Wales perceived distances differently and therefore saw distance as a lesser/greater obstacle to migration.

Therefore, whereas Table 1 includes only single parameters for each predictor of migration flows—implicitly assuming that each independent variable had the same effect on migration flows across England and Wales–Eq 3 describes the model that was run for *each RSD of origin separately*, thereby producing origin-specific parameters [115]. As such, the origin-specific determinants described in Table 1 become redundant and are therefore excluded.


$$\begin{array}{cc} \lambda_{ij}= & \text{exp}(\beta_{0}+\beta_{1}I_{j}+\beta_{2}\;\text{ln}\;M_{j}+\beta_{3}\;\text{ln}\;P_{j}+\beta_{4}\;\text{ln}\;D_{j}+\beta_{5}\;\text{ln}\;\left(P_{j\;\left(x-10\right)}/P_{j\;\left(x-20\right)}\right)+\beta_{6}\;\text{ln}\;H_{j}\\ & +\beta_{7}\;\text{ln}\;R_{j}+\beta_{8}\;\text{ln}\;U_{j}+\beta_{9}\;\left(U_{j}\times Z_{ij}\right)+\beta_{10}\;\left(U_{j}\times \text{ln}\;d_{ij}\right)+\beta_{11}\;\left(Z_{ij}\times \text{ln}\;d_{ij}\right)) \end{array}$$ (3)

Eq 3 consequently differs from the model presented in Table 1 slightly. First, whereas Table 1 shows an unconstrained model with both origin- and destination-specific parameters, Eq 3 includes only destination-specific parameters in addition to both the distance migrated (*d*_*ij*_) and whether a boundary between two cultural provincial was crossed or not (*Z*_*ij*_). Second, Table 1 includes *T*_*j*_ as a categorical predictor, which includes London as a separate category to other urban destinations, while variable *U*_*j*_ in Eq 3 only distinguishes between urban and rural destinations. In Wilson’s family of spatial interaction models, destination-constrained models include a fixed effect for the destination, ensuring that the total number of in-migrants to each destination equals that observed [8]. As Eq 3 produces origin-specific parameters, adding in a destination-specific category—such as London—would create a destination-constrained model for each origin, such that equation 5 would perfectly predict the number of migrants from each RSD to London. As this would over-specify the model, parameter *T*_*j*_ was dropped in favour of *U*_*j*_.

**Fig 10:**
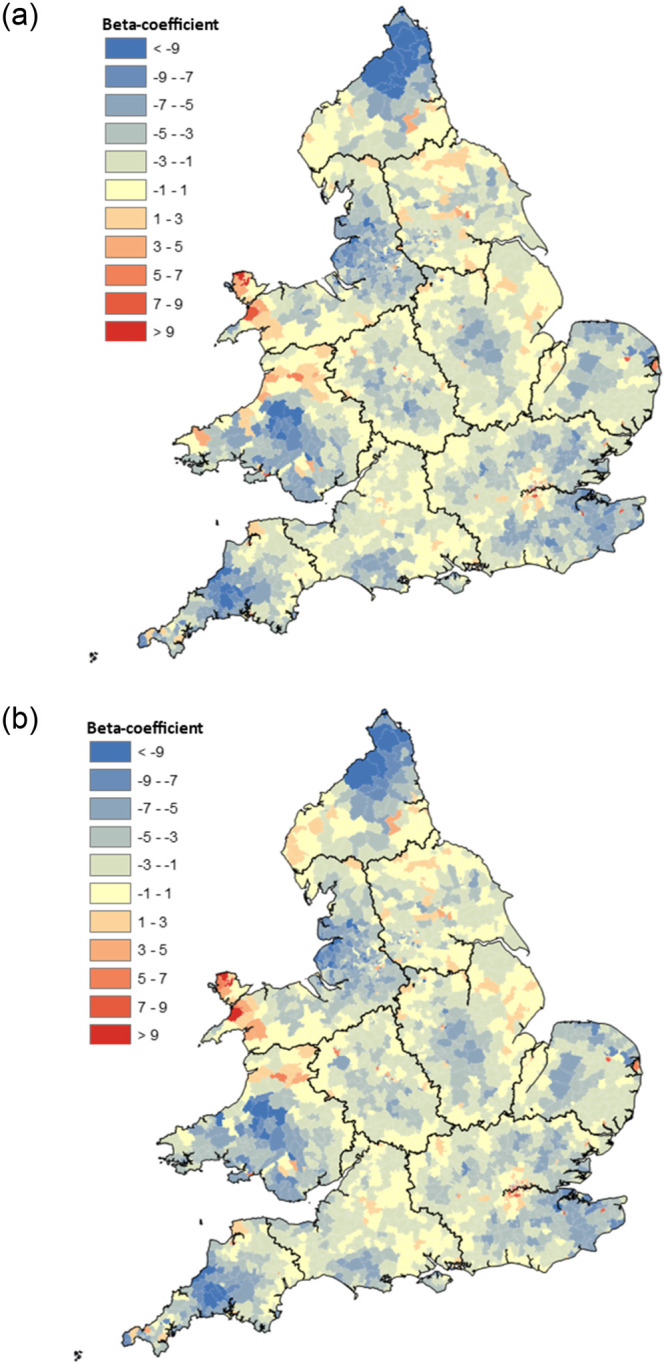
Origin-specific beta-coefficients of interaction effect (*U*_*j*_ × *Z*_*ij*_) from Eq 3. Effect of provincial boundaries on migration flows to rural/urban destinations. England & Wales 1851–1911. **a) Rural Destination, b) Urban Destination.** Source: Author’s analysis based on data from UK Data Service SN 7481 (Schürer, 2019).

Firstly however, it is worth noting that by producing 2,110 origin-specific models, the total variance of *all* migration paths as measured by the pseudo-*R*^2^ –improved dramatically to 0.857. As this suggests that parameters did indeed have a differential effect on individuals’ propensity to migrate, it would be appropriate to determine whether provincial boundaries acted as a greater brake on some migrants’ choice of destination than others. Fig 10 maps the beta-coefficients of the interaction effect (*U*_*j*_ × *Z*_*ij*_), which shows the effect that crossing a provincial boundary had on the volume of migration—dependent on whether the destination was a rural or urban one.

As expected, while boundaries had an almost universal negative effect on the number of migrants, regardless of whether their destination was urban or rural, when overlaid with the provincial boundaries identified in this article, Fig 10 shows an apparent weakening of this effect nearer the boundary of each province. In other words, boundaries do not appear to have been distance-neutral barriers to migration, but rather, the further one had been born from such a boundary, the greater an obstacle to migration they became. As distance has already been explicitly included in the model, it is unlikely that such boundaries represent an uncaptured distance-decay effect and instead, perhaps suggests that migrants born near such boundaries had more exposure to other provincial cultures, thereby reducing—but not altogether removing—their reticence to migrate across cultural borders [116, 117].

**Fig 11:**
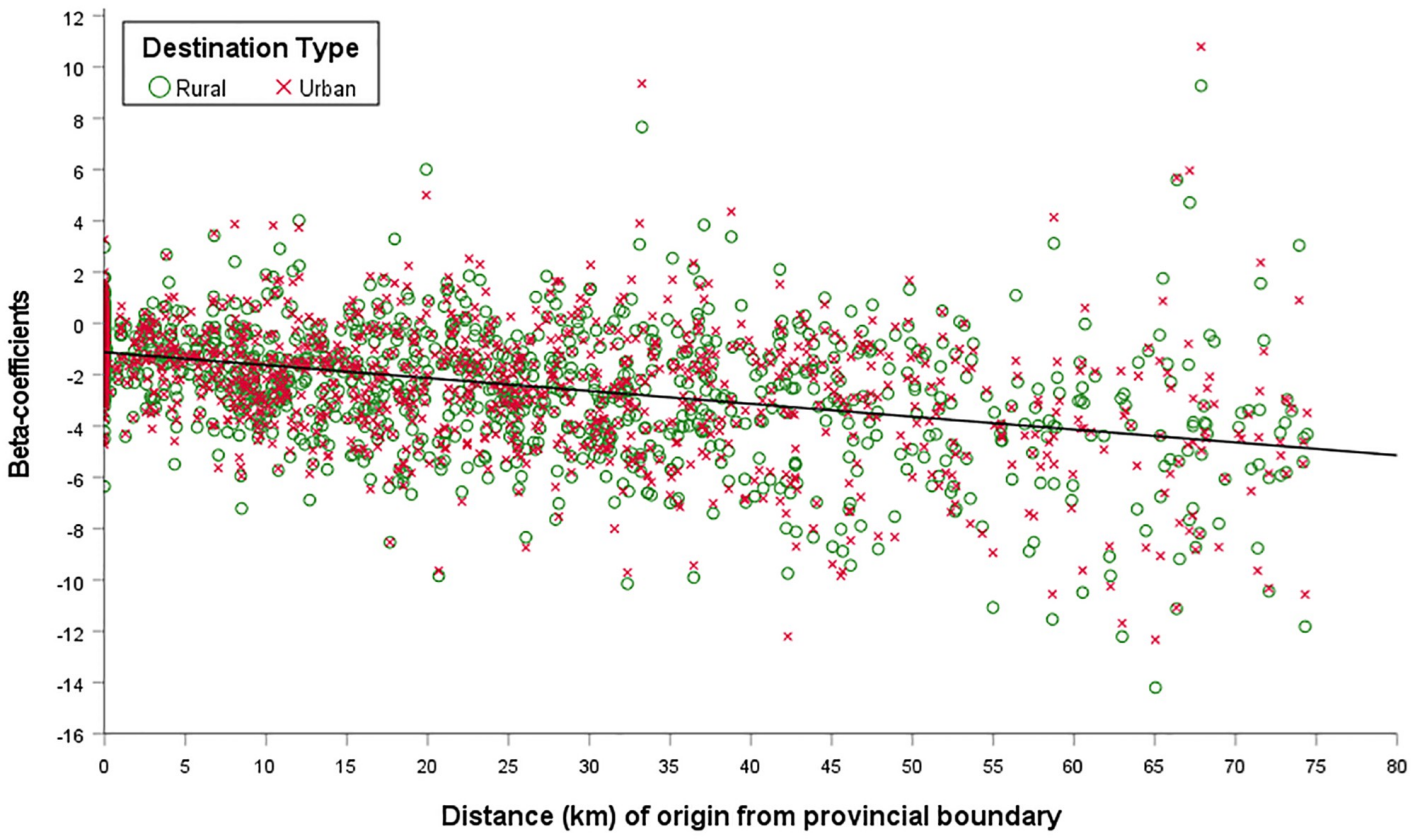
Relationship between the distance of a rural origin from a provincial boundary and the corresponding origin-specific boundary effect on migration flows. By destination type. England & Wales 1851–1911. Source: Author’s analysis based on data from UK Data Service SN 7481 (Schürer, 2019).

Confirming this, Fig 11 shows a scatterplot of the origin-specific parameter estimates of the interaction (*U*_*j*_ × *Z*_*ij*_) on migration flows, against the distance of each origin from a boundary, distinguishing between rural and urban destinations [16, 88]. Several observations are of note. Firstly, although there is not a statistically significant difference between rural and urban destinations, the overall correlation is both significant and relatively strong, with a p-value of <0.01 and an adjusted *R*^2^ of 0.155. Secondly, the relationship between distances from a provincial boundary and the strength of the effect that crossing a provincial boundary had on the volume of migration is clear from the line of best-fit *Y*_*i*_ = −1.13 − 0.05*x*_*i*_ + *ε*_*i*_. Not only does the coefficient of *x*_*i*_ show that the further that migrants had been born from a provincial boundary, the more the boundary inhibited migration flows across it, but the negative *y*-intercept of -1.13, shows that boundaries inhibited migration across them at *any* distance.

This interpretation reiterates that made of Fig 7, which showed that less than half of migrants born in an RSD bordering another province migrated across such a boundary. Clearly, cultural boundaries had an important—but overlooked—effect, not only on migration *per se*, but specifically on the transfer of rural to urban labour and by extension, the speed of urbanisation.

## 5. Concluding remarks

This article has sought to identify and elucidate two interrelated processes in British economic geography. Firstly, by applying a community detection algorithm to migration data, it has been possible to not only identify long-standing barriers to human interaction, but provide an empirical validation for the presence, location, and spatial limits of cultural provinces, long posited by Fawcett, Gilbert, and Phythian-Adams [40–44]. Having identified the extent of these cultural provinces, a spatial interaction model (SIM) was employed to quantify their impact on migration, further evidencing the significance of these areas as discrete regions in which distinct—and largely separate—groups interact, therefore representing distinct communities. Analysing the interaction effect of moving between cultural provinces and migrants’ type of origin and destination in an unconstrained SIM as shown in Table 1, it is apparent that cultural boundaries limited all types of migration, including rural to urban moves. Therefore, cultural boundaries not only added to the friction of distance, but in so doing, delayed the pace at which Britain urbanised. However, while cultural boundaries did indeed act as barriers, such boundaries did not inhibit all migrants equally.

Rather, when modelling migration from each RSD of origin separately, the parameter estimates show that while the effect of boundaries on limiting migration flows was not dependent on migrants’ choice of destination, it was dependent on how far rural-born migrants had been born from such a boundary. While boundaries limited the number of all migrants crossing them, their effect appears to be stronger the further migrants had been born from one. Indeed, Fig 11 shows that where migrants had been born in an RSD adjoining a provincial boundary the coefficient effect of boundaries on migration flows was -1.13, rising to -5.13 where migrants had been born 80km from the provincial border.

This article has therefore not only identified the most statistically significant breaks in the network of human interaction across a sixty-year period in England and Wales, but also demonstrated that such breaks were meaningful, as even once other factors are accounted for, the boundaries identified inhibited migration across them, slowing the pace of urbanisation. While it has therefore demonstrated that the cultural geographies of identity and belonging were significant determinants of the economic geographies of migration, it has only intimated that such barriers to human movement represented bottlenecks in the labour market which might explain—at least in part—persistent rural/urban wage disparities, as the rural labour market was unable to fully satisfy the seemingly insatiable urban demand for labour in the second half of the nineteenth century.

To return to the metaphor employed at the outset, the stream moving the pool of rural labour to the urban pool had an insufficient carrying capacity, exacerbated by cultural barriers partially damming the flow. Only once comprehensive, individual-level wage estimates have been produced for the period 1851–1911, will it be possible to analyse the extent to which migration did indeed narrow the wage gap between the places migrated from and to, and whether the barriers between cultural provinces did—as has been hypothesised here—limit the ability of the labour market to clear. Or, as supposed by Long, Boyer and Hatton, the labour market remained in perpetual disequilibrium because the price signal—i.e. wages—did not work to efficiently redistribute labour from the least- to the most-productive employment [26, 29]. Whatever the truth may be, it is hoped that this article has at the very least, raised the possibility that questions of economic inequality may have their answers in longstanding cultural and behavioural norms and therefore deserves serious consideration as a determinant of economic change in both the past and present.
